# Mechanistic Action of Cell Cycle Arrest and Intrinsic Apoptosis via Inhibiting Akt/mTOR and Activation of p38-MAPK Signaling Pathways in Hep3B Liver Cancer Cells by Prunetrin—A Flavonoid with Therapeutic Potential

**DOI:** 10.3390/nu15153407

**Published:** 2023-07-31

**Authors:** Abuyaseer Abusaliya, Se Hyo Jeong, Pritam Bhagwan Bhosale, Hun Hwan Kim, Min Yeong Park, Eunhye Kim, Chung Kil Won, Kwang Il Park, Jeong Doo Heo, Hyun Wook Kim, Meejung Ahn, Je Kyung Seong, Gon Sup Kim

**Affiliations:** 1 Department of Veterinary Medicine, Research Institute of Life Science, Gyeongsang National University, 501 Jinju-daero, Jinju 52828, Republic of Korea; yaseerbiotech21@gmail.com (A.A.);; 2 Biological Resources Research Group, Gyeongnam Department of Environment Toxicology and Chemistry, Korea Institute of Toxicology, 17 Jegok-gil, Jinju 52834, Republic of Korea; 3 Division of Animal Bioscience & Integrated Biotechnology, Jinju 52725, Republic of Korea; 4 Department of Animal Science, College of Life Science, Sangji University, Wonju 26339, Republic of Korea; 5 Laboratory of Developmental Biology and Genomics, BK21 PLUS Program for Creative Veterinary Science Research, Research Institute for Veterinary Science, College of Veterinary Medicine, Seoul National University, Seoul 08826, Republic of Korea

**Keywords:** flavonoids, liver cancer, Hep3B cells, cell cycle arrest, apoptosis

## Abstract

Hepatocellular carcinoma (HCC) has a poor prognosis and a low survival rate. Drugs without side effects are desperately needed since chemotherapy has a negative effect on the host cells. Previous research has firmly established that plant-based compounds have significant bioactivities without a negative impact on the host. Flavonoids, in particular, are a class of compounds with both anti-inflammatory and anti-cancer properties. Prunetrin (PUR) is a glycosyloxyisoflavone (Prunetin 4′-O-glucoside) derived from *Prunus* sp., and its other form, called prunetin, showed optimistic results in an anti-cancerous study. Hence, we aimed to discover the anti-cancer ability of prunetrin in liver cancer Hep3B cells. Our cytotoxicity results showed that PUR can decrease cell viability. The colony formation assay confirms this strongly and correlates with cell cytotoxicity results. Prunetrin, in a dose-dependent manner, arrested the cell cycle in the G2/M phase and decreased the expression of cyclin proteins such as Cyclin B1, CDK1/CDC2, and CDC25c. Prunetrin treatment also promoted the strong cleavage of two important apoptotic hallmark proteins called PARP and caspase-3. It also confirms that apoptosis occurs through the mitochondrial pathway through increased expression of cleaved caspase-9 and increased levels of the pro-apoptotic protein Bak. Bak was significantly increased with the declining expression of the anti-apoptotic protein Bcl-xL. Next, it inhibits the mTOR/AKT signaling pathways, proving that prunetrin includes apoptosis and decreases cell viability by suppressing these pathways. Further, it was also observed that the activation of p38-MAPK was dose-dependent. Taken together, they provide evidence that prunetrin has an anti-cancerous ability in Hep3B liver cancer cells by arresting the cell cycle via p38 and inhibiting mTOR/AKT.

## 1. Introduction

Hepatocellular carcinoma (HCC) represents approximately 90% of primary liver cancers and is considered a major global health concern [1]. According to the WHO’s GLOBOCAN 2020 report, a roughly 28.4 million-case registry is anticipated in 2040, and the report predicts that liver cancer is the fourth leading cause of death among other cancers [2]. Liver cancer is a serious medical condition that affects thousands of individuals every year. The American Cancer Society reports that the five-year survival rate for people with liver cancer is only 20%, illustrating the severity of this disease. Furthermore, the prognosis is even more unfavorable when cancer spreads to distant sites, with a 4% five-year survival rate. Primary liver cancer, which accounts for the majority of cases, has a very low 5-year survival rate of only 5–9% [3,4,5]. Despite advances in treatment options such as chemotherapy (including trans-arterial embolization (TAE) and conventional trans-arterial chemoembolization (TACE)), radiation therapy (selective internal radiation therapy (SIRT)), surgery, and systemic treatment like immunotherapy, the overall survival rate of liver cancer patients remains poor. The main obstacle to treatment is that hepatocellular carcinoma (HCC) is diagnosed in its advanced stages. Only a small proportion of patients are suitable for curative surgical interventions like resection or transplantation [6].

Currently, cisplatin, sorafenib, and lenvatinib combined with transarterial chemoembolization are used to treat cancer based on the tumor stage of HCC. Cisplatin and sorafenib are cytotoxic agents and tyrosine kinase inhibitors, respectively. Regrettably, the reported studies indicate that cisplatin has an adverse effect on the host cells and causes drug resistance [7,8,9]. Hence, in recent years, natural- or plant-based compounds with anti-cancerous effects have gained attention due to their lower side effects and potential to inhibit cancer by modulating cell signaling, arresting the cell cycle, initiating caspase cascades, and treating adverse effects caused by chemotherapy. 

Targeting apoptosis is a beneficial strategy for developing new cancer therapeutics because apoptosis is a process that results in cell death to sustain homeostasis. Generally, apoptosis occurs via either the extrinsic pathway (death receptor pathway), the intrinsic pathway (mitochondrial pathway), or both [10]. In the extrinsic pathway, cell death happens from external stimuli that bind to death receptors (Fas, TNF, etc.) on the cell surface. On the other hand, stimuli inside the cell, mainly involving mitochondria, result in DNA damage, caspase cascade activation, and cell death [11]. Similarly, mitogen-activated protein kinase (MAPK) and AKT serine-threonine kinase/mammalian target of rapamycin (Akt/mTOR) play a key role in cell proliferation in cancer cells [12]. The MAPK pathway is a signaling cascade that plays an essential role in various cellular processes, including cell proliferation, differentiation, apoptosis, and stress response [13]. Similarly, the Akt/mTOR pathway is a crucial signaling network involved in regulating cell growth and survival, and its dysregulation is linked to the growth and development of cancer [14].

Flavonoids, bioactive compounds found in plants, have gained attention for their potential anti-cancer properties [15]. Several studies have reported that flavonoids can reduce the risk of developing various types of cancer, including gastric [16], breast [17], prostate [18], and colorectal [19]. Prunetrin (Prunetin 4′-O-glucoside) is a glycosyloxyisoflavone derived from *Prunus sp.* The ancestor form of prunetrin is called prunetin, which binds to RIPK3 and induces necroptotic cell death in gastric cancer [20]. Also, prunetin’s glycosidic form, called prunetinoside, showed a therapeutic effect by binding to targets in gastric cancer [21]. It showed potential as an anti-inflammatory drug by reducing inflammation by activating NF-κB and MAPK in macrophage cells [22]. However, the effect of prunetrin on HCC lacks literature. Thus, we aimed to explore the anti-cancerous ability of PUR and its mechanism of action in hepatocellular carcinoma (Hep3B) cells.

## 2. Materials and Methods

### 2.1. Cell Line and Maintenance

Hep3B cells obtained from ATCC (Cat# HB-8064) were cultured in DMEM with 10% FBS (Gibco; Thermo Fisher Scientific, Waltham, MA, USA), 100 g/mL streptomycin, and 100 U/mL penicillin (Gibco; Thermo Fisher Scientific). The cells were kept at 37 °C in a humidified environment with 5% CO_2_. The purity of prunetrin is ≥98%.

### 2.2. Cytotoxicity Test

Cell proliferation assay was used to determine the cytotoxic ability of the compound. Cells were plated at a cell volume of 1 × 10^4^ cells/well in a 96-well plate, then treated at 37 °C for 24 h with a progressive concentration of PUR (0.5, 1, 2, 4, 6, 8, 10, 20, 30, 40, and 50 μM). 3-(4,5-dimethylthiazol-2-yl)-2,5-diphenyl tetrazolium bromide (MTT) (Duchefa Biochemie, Haarlem, The Netherlands) solution (10 μL; 5 mg/mL) was added to the wells after the treatment and incubated for 4 h. After incubation, the MTT solution was fully discarded, and dimethyl sulfoxide (DMSO) was added to dissolve the insoluble formazan crystals. The absorbance of the solution in the plates was then read using a microplate photometer at 590 nm wavelength (Thermo Scientific).

### 2.3. Colony Formation Assay

Colony formation assay was performed to study the ability of cells to grow into a colony and its survival. Cells were seeded at 1 × 10^5^ density in 6 well plates, and the cells were treated with PUR (5, 10, 20, 30, and 40 μM) and kept in incubation for 2 weeks. After that, plates were washed with phosphate-buffered saline (PBS) and fixed with 4% paraformaldehyde for 30 min at RT, and then the cells were stained with 0.6% Giemsa stain and washed to remove the excess stain. Then the cells were picturized, and the colonies were counted and analyzed by the ImageJ software V 1.52a program (U.S. National Institutes of Health, Bethesda, MD, USA).

### 2.4. DAPI Staining

DAPI staining was used to study the nuclear morphology. Cells were seeded at 1 × 10^5^ density in 6-well plates, and the cells were treated with PUR (10, 20, 30, and 40 μM) for 24 h. After washing with PBS, fixed cells were stained with 4′,6-diamidino-2-phenylindole (DAPI; Vectashield H-1500; Vector Laboratories, Burlingame, CA, USA). Fluorescence microscopy (EVOS^®^, Life Technologies, Darmstadt, Germany) was used to study the nuclear morphology of the cells.

### 2.5. Cell Cycle Analysis

Cell cycle analysis detected cell population accumulation at different cell cycle stages. Hep3B cells were seeded at a cell density of 5 × 10^4^ cells/plate in a 60π plate and treated with the different concentrations of PUR (10, 20, and 40 μM) for 24 h. Then, the cells were given a cold PBS wash, followed by trypsin addition to detach the cells, collected in conical tubes, and centrifuged. Then, ice-cold PBS was added to the pellet, and 70% ethanol with PBS was added and kept at −20 °C for about 1 h. Then, the tubes were centrifuged and resuspended in 1X solution containing propidium iodide (PI) (Sigma-Aldrich, St. Louis, MO, USA), PBS, and RNase A. This mixer setup was incubated in RT for 15 min at dark. After incubation, the cells were sorted by flow cytometry using FACSVerse^TM^ (BD Biosciences, Franklin Lakes, NJ, USA). The acquired data were evaluated by ModFit LT 6.0 software (Verity Software House, Topsham, ME, USA).

### 2.6. Double Staining

Double staining helps to find the different cell death modes. Hep3B cells were seeded at a cell density of 5 × 10^4^ cells/plate in a 60π plate, followed by PUR (10, 20, and 40 μM) treatment for 24 h. Then, the cells were given a cold PBS wash, and trypsin was added to detach the cells. The collected cells were placed in conical tubes and centrifuged, and the pellet obtained was washed with PBS and again centrifuged. Then the pellet was resuspended with 1X binding buffer. According to the manufacturer’s instructions, APC/Annexin V and PI were added from the apoptosis detection kit (BD Biosciences, San Diego, CA, USA). Then the cells were sorted (10,000 events at moderate speed) in the flow cytometry FACSVerse^TM^ (BD Biosciences, Franklin Lakes, NJ, USA).

### 2.7. Western Blotting

Cells were planted at a cell density of 5 × 10^4^ cells/plate in a 60π plate to evaluate protein expression. Cells were treated for 1 h with 10, 20, and 40μM PUR for 24 h at 37 °C. Cells were subsequently collected and lysed with radioimmunoprecipitation assay (RIPA) buffer (iNtRON Biotechnology, Seongnam, Republic of Korea), which included [50 mM Tris-HCl (pH 8.0), 0.5% sodium deoxycholate, 1 mM EDTA, 150 mM NaCl, 0.1 SDS, and 1% NP40]. The protein concentration was then calculated using a Pierce^TM^ BCA protein assay kit (Thermo Scientific). SDS-PAGE was used to separate the proteins (10 g). The gel was then transferred to PVDF membranes using a semi-dry transfer unit (Atto Corporation, Tokyo). After blocking the membranes for 1–2 h at room temperature with 5% bovine serum albumin (BSA, Irving, TX, USA; Thermo Fisher Scientific), the membranes were incubated overnight at 4 °C with diluted (1:1000) primary antibodies. Then, the membranes were washed with 1X TBST solution before being incubated with the appropriate horseradish peroxidase-conjugated secondary antibody (1:5000) for 2 h at room temperature. The blots were then washed with 1X TBST and developed using an electrochemiluminescence (ECL) detection system (Bio-Rad Laboratory, Hercules, CA, USA). ImageJ software (U.S. National Institutes of Health, Bethesda, MD, USA) was used to evaluate protein densitometry.

### 2.8. Statistical Analysis

Statistical analysis was performed by Dunnett’s Multiple Comparison Test. GraphPad Prism V.5.01 software was used to perform the analysis. A *p*-value of <0.05 was considered statistically significant.

## 3. Results

### 3.1. Prunetrin Induces Cytotoxicity in Hep3B Cells

**Figure 1:**
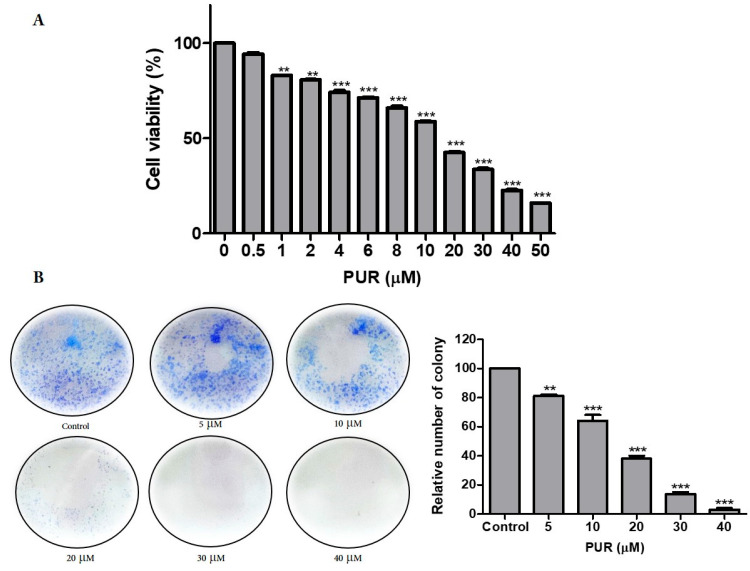
Effect of PUR on Hep3B proliferation. (**A**) Cell cytotoxicity of Hep3B cells; (**B**) Colony formation assay. The cell viability was measured by using MTT after being treated for 24 h with the different concentrations of PUR (0, 0.5, 1, 2, 4, 6, 8, 10, 20, 40, and 50 μM). For colony formation assay, cells were treated with 5, 10, 20, 30, and 40 μM PUR for 2 weeks. The results are presented as mean ± standard error of the mean (SEM). ** = *p* < 0.01, and *** = *p* < 0.001.

To determine the cytotoxic ability of prunetrin in Hep3B cells, MTT assays were performed. As shown in Figure 1A, prunetrin can induce cytotoxicity in cancer cells and significantly inhibit cell proliferation dose-dependently, and the cell viability percentage was below 50 from 20 μM to 50 μM. We further performed a colony formation assay. As shown in Figure 1B, colony formation was significantly reduced when Hep3B cells were treated with prunetrin, especially in higher concentrations. Hence, the three treatment concentrations (10, 20, and 40 μM) for further experiments were selected. These results suggest that the compound prunetrin can induce cytotoxicity and decrease cell proliferation in Hep3B cells.

### 3.2. Morphological Assessment of Hep3B Cells Treated with PUR

**Figure 2:**
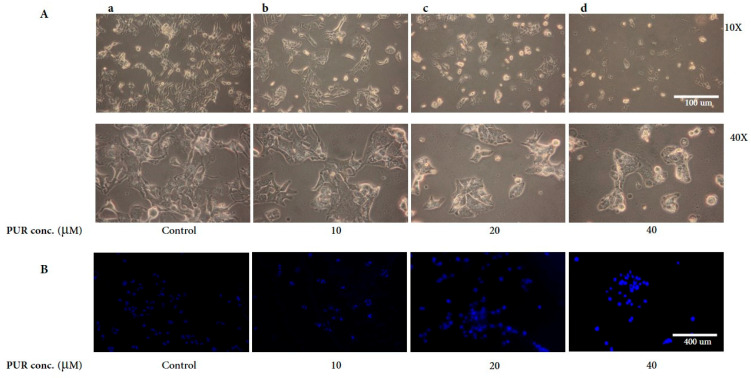
Morphological changes associated with prunetrin treatment. (**A**) Light microscopic observation of Hep3B cells upon PUR treatment; (**B**) DAPI fluorescent observation for the nuclear. For morphological assessment, cells were treated with PUR (10, 20, and 40 μM) for 24 h, and then cells were observed under an inverted microscope. For DAPI staining, cells were treated with PUR (10, 20, and 40 μM) for 24 h, then stained with DAPI dye and observed under a fluorescence microscope.

Cells were treated with PUR (10, 20, and 40 μM) to observe the morphological alternations and observed under the light microscope. Compared to the control cells (Figure 2A(a)), PUR-treated cells tend to have cell death morphological features, including shrinkage, condensation, and cytoplasmic blebbing (Figure 2A(b–d)). On the other hand, the cell counts decreased dose-dependently, and detached cells were high in PUR-treated cells. Nuclear condensation is the core indication of apoptosis in cancer cells. Hence, to determine whether PUR causes nuclear condensation, we performed DAPI staining to observe the nuclear morphology. As a result, there was a darker, intense blue coloration in the higher dose than in the control group (Figure 2B), indicating nuclear condensation. These results correlate with the cytotoxicity data and demonstrate that PUR-treated cells undergo the cell death process.

### 3.3. Prunetrin Induces Cell Cycle Arrest in Hep3B Cells

**Figure 3:**
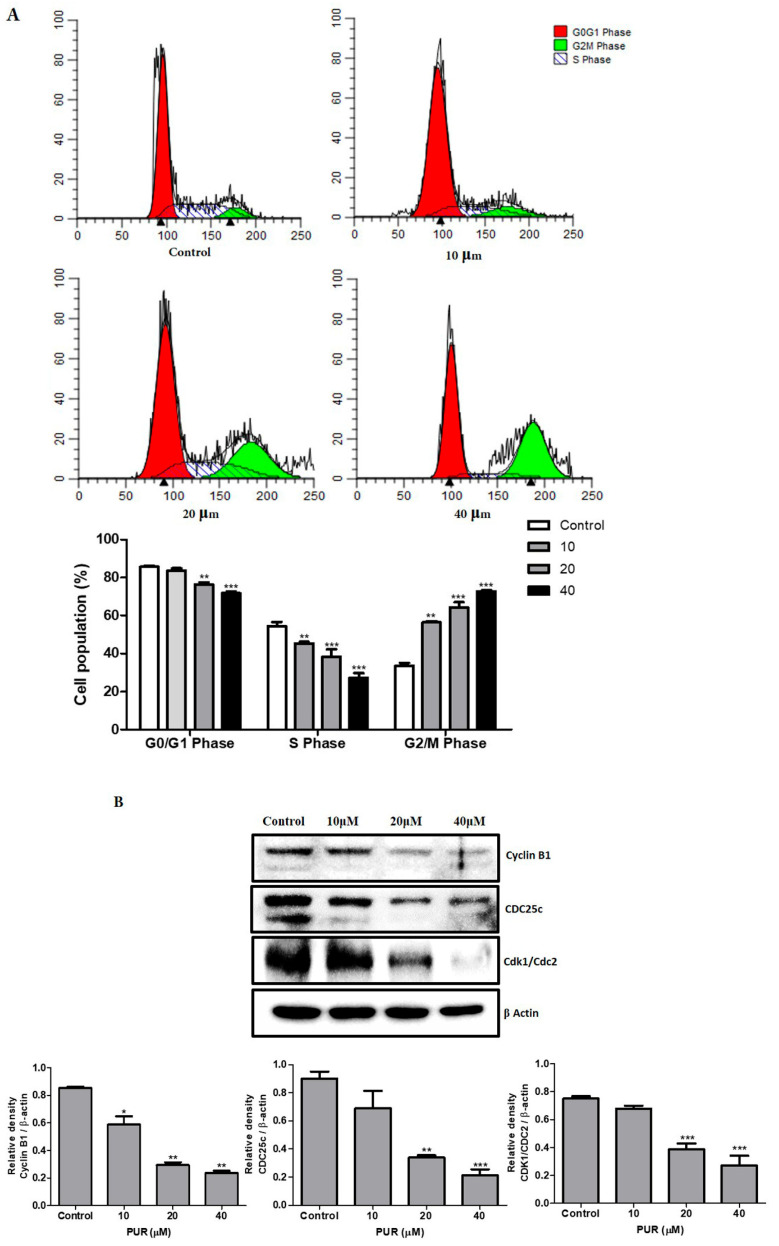
Effect of PUR in cell cycle and expression of cell cycle-related proteins. (**A**) Flow cytometry analysis of cell cycle; (**B**) protein expression analysis by Western blotting. The cells were treated with PUR (10, 20, and 40 μM) for 24 h, and the cell cycle distribution was determined with PI using flow cytometry. For the Western blot, the protein was isolated from the cells after treatment, and densitometry was used to determine the expression levels using the SEM of three independent values. The results are presented as mean ± standard error of the mean (SEM). * = *p* < 0.05, ** = *p* < 0.01, and *** = *p* < 0.001. [CDK1/CDC2—cyclin-dependent protein kinase-1/cell division control-2; CDC25c—cell division cycle 25c].

To determine the influence of prunetrin on the cell cycle, Hep3B cells underwent 24 h of PUR treatment, and the cell cycle distribution analysis was carried out using PI by flow cytometry. As presented in Figure 3A, the cell population was highly accumulated at the G2/M phase in prunetrin-treated cells compared to the control cells. To explore deeply the cell cycle arrest at G2/M phase, the key cell cycle regulating proteins like cyclin-dependent protein kinase-1/cell division control-2 (CDK1/CDC2), cell division cycle 25c (CDC25c), and cyclin B1 expression were analyzed by Western blotting. The results show that all three protein expressions decreased dose-dependently (Figure 3B). This suggests that prunetrin inhibits cell proliferation by arresting the cell cycle at the G2/M phase by decreasing the expression of cell cycle-regulating proteins.

### 3.4. Prunetrin Induces Apoptosis and Increases the Expression of Apoptotic Hallmark Proteins

**Figure 4:**
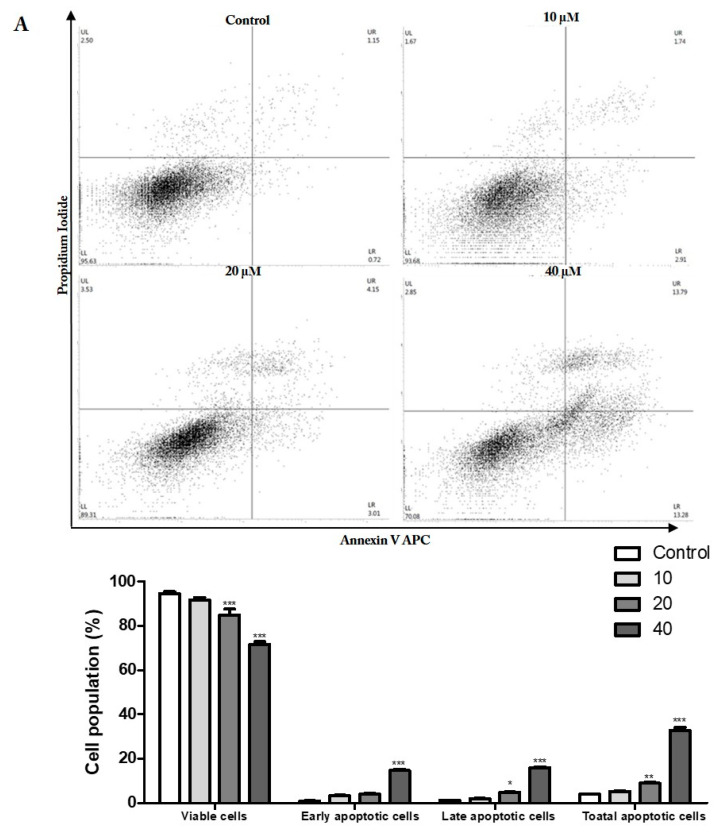

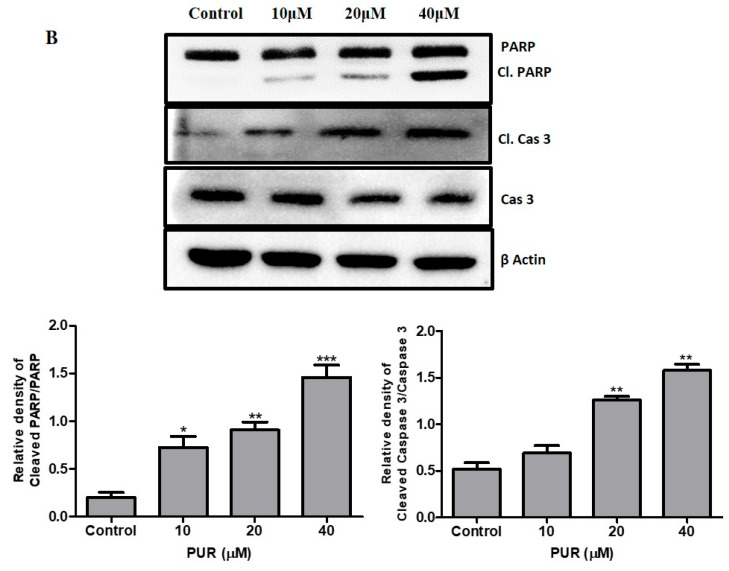
Effect of PUR on apoptotic hallmarks. (**A**) Apoptosis analysis by flow cytometry; (**B**) Western blot analysis of cell cycle-related proteins. The cells were treated with PUR (10, 20, and 40 μM) for 24 h and then stained with Allophycocyanin (APC)/Annexin V and propidium iodide (PI). The apoptotic distribution was determined using flow cytometry. Then, isolated proteins were used for Western blot, and densitometry was used to determine the expression levels using the SEM of three independent values. The results are presented as the mean ± standard error of the mean (SEM). * = *p* < 0.05, ** = *p* < 0.01, and *** = *p* < 0.001. [PARP—poly (ADP-ribose) polymerase; Cl. PAPR—cleaved poly (ADP-ribose) polymerase (PARP); Cl. Cas 3—cleaved caspase 3; Cas 3—caspase 3].

To determine whether prunetrin induces apoptotic-mediated cell death, we analyzed it with Annexin V/PI using flow cytometry. With the 24 h PUR treatment, there is an increased cell population in the total apoptotic cells. At the higher dose of PUR, cells are grouped into early and late apoptotic populations, from 0.72% and 1.15% to 13.28% and 13.79%, respectively, to compare with the control group (Figure 4A). In particular, the total apoptotic cells increased to over three times the amount in the higher dose. To further confirm this, Western blotting was performed to check the expression of the apoptotic hallmark proteins cleaved poly (ADP-ribose) polymerase (PARP) and cleaved caspase 3. As intended, there is a robust threefold increase in cleaved PARP at the higher dose and a significant increase in cleaved caspase 3 (Figure 4B). These protein expressions were increased in the PUR treatment dose-dependently, indicating that the PUR induces late apoptotic-mediated cell death in Hep3B cells.

### 3.5. Prunetrin Activates an Intrinsic Apoptotic Pathway in Hep3B Cells

**Figure 5:**
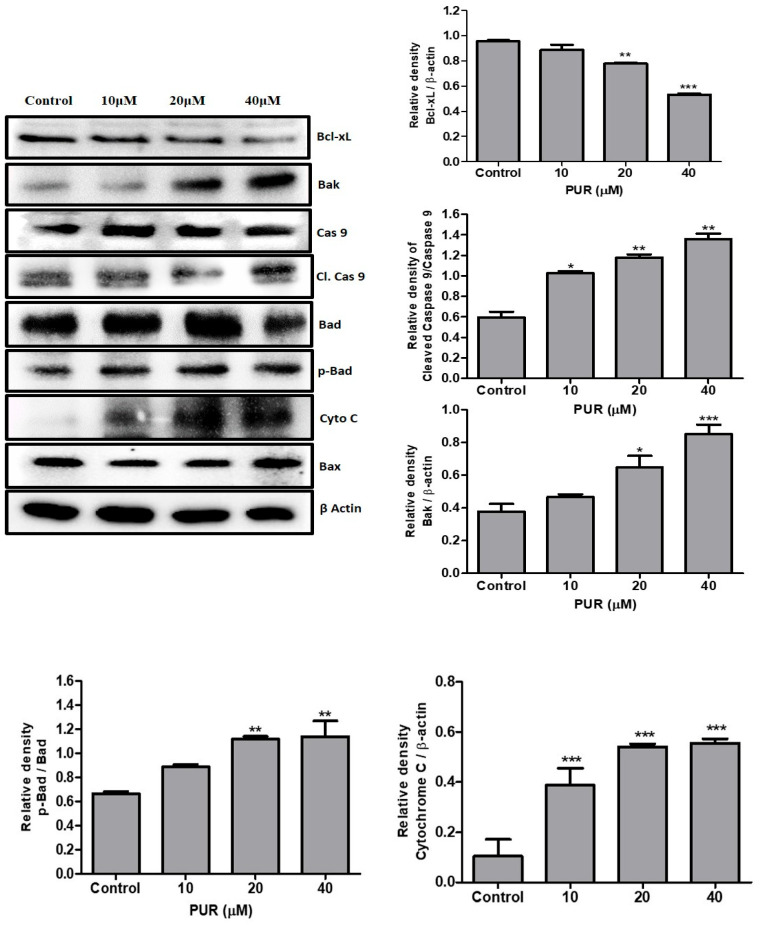
Effect of PUR on intrinsic apoptotic proteins. The cells were treated with PUR (10, 20, and 40 μM) for 24 h, and isolated proteins were used for Western blotting. Densitometry was used to determine the expression levels using the SEM of three independent values. The results are presented as the mean ± standard error of the mean (SEM). * = *p* < 0.05, ** = *p* < 0.01, and *** = *p* < 0.001. [Bcl-2—B-cell lymphoma 2; Bak—Bcl-2 homologous antagonist killer; Bad—Bcl-2-associated death; Bax—Bcl-2-associated X protein; cas 9—caspase 9; cl. Cas 9—cleaved caspase 9; pBad—phospho Bad; cyto C—cytochrome C].

To confirm the type of apoptosis that PUR arbitrates initially, Western blotting was used to analyze mitochondrial-mediated proteins, namely B-cell lymphoma 2 (Bcl-2), Bcl-2 homologous antagonist killer (Bak), Bcl-2-associated death (Bad), and Bcl-2-associated X protein (Bax). As shown in Figure 5, the anti-apoptotic protein Bcl-2 was significantly decreased with higher expression of the pro-apoptotic protein Bak in a dose-dependent manner. Also, the pBad/Bad ratio increased significantly, while Bax had no significant change. Plus, the intrinsic apoptotic regulatory protein cleaved caspase-9 was notably increased in the higher dose. These findings suggest that prunetrin induces apoptosis in Hep3B cells via an intrinsic apoptotic pathway by upregulating pro-apoptotic proteins (Bak and pBad/Bad) and a decline in anti-apoptotic proteins (Bcl-2) with caspase-9 activation. 

### 3.6. Prunetrin Does Not Induce Extrinsic Apoptosis and Other Cell Death Modes in Hep3B Cells

**Figure 6:**
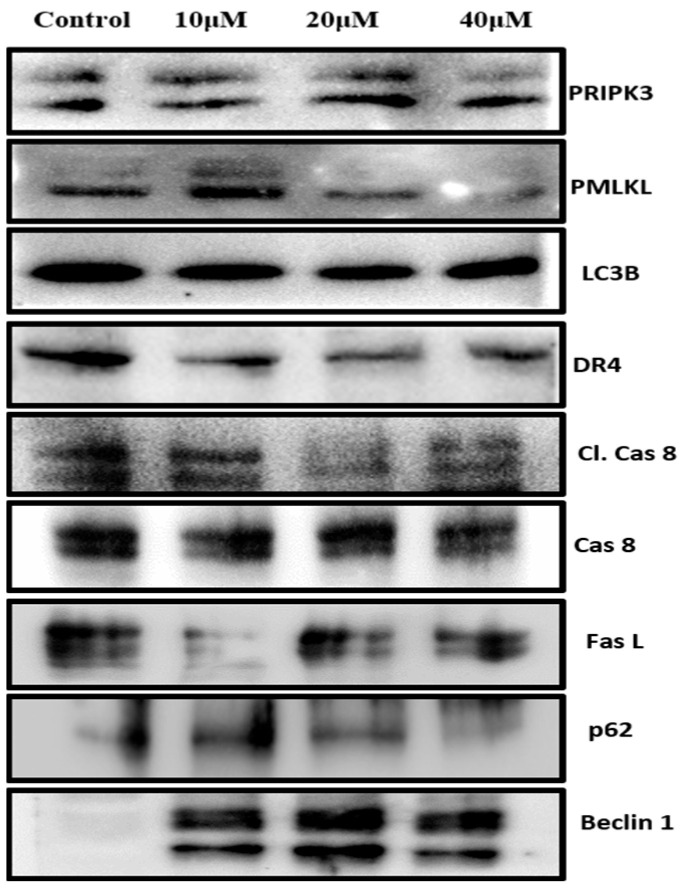
PUR does not induce other modes of cell death. The cells were treated with PUR (10, 20, and 40 μM) for 24 h, and then isolated proteins were used for Western blotting. [PRIPK3—phospho-receptor-interacting serine/threonine-protein kinase 3; PMLKL—phospho-mixed lineage kinase domain-like; DR4—Death receptor-4; Cl. Cas 8—cleaved caspase 8; cas 8—caspase 8; FasL—Fas Ligand].

To confirm that PUR only induces the intrinsic apoptotic pathway and no other mode of cell death is involved, we examined the expressions of the key proteins involved in the extrinsic cell death pathway, necrosis, and autophagy. Specifically, we analyzed the levels of cleaved caspase 8, Fas ligand (FasL), Death receptor-4 (DR4), phospho-receptor-interacting serine/threonine-protein kinase 3 (RIPK3), phospho-mixed lineage kinase domain-like (pMLKL), Beclin 1, p62, and LC3B. Even at higher doses, there are no alterations in phosphorylation or expression (Figure 6). These results strongly suggest that PUR did not induce other modes of cell death than the intrinsic pathway of cell death in Hep3B cells.

### 3.7. Prunetrin Inhibits AKT/mTOR Signaling Pathway

**Figure 7:**
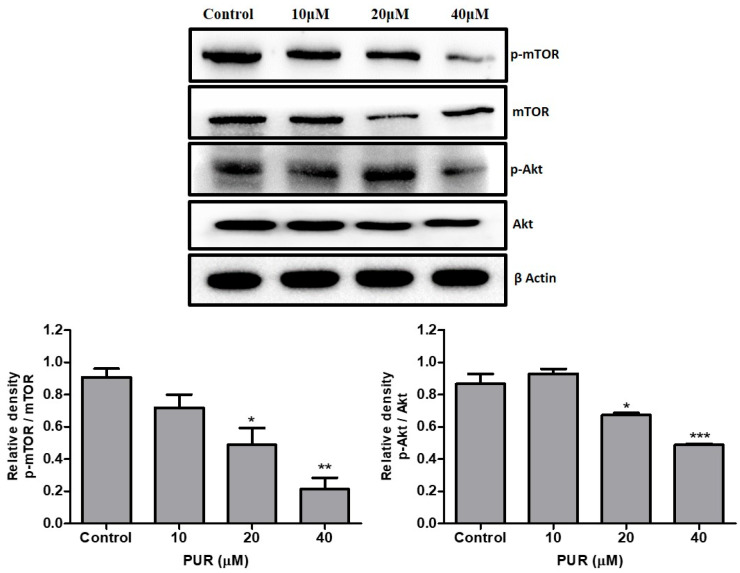
Effect of PUR on the AKT/mTOR signaling pathway. The cells were treated with PUR (10, 20, and 40 μM) for 24 h, and isolated proteins were used for Western blotting. Densitometry was used to determine the expression levels using the SEM of three independent values. The results are presented as the mean ± standard error of the mean (SEM). * = *p* < 0.05, ** = *p* < 0.01, and *** = *p* < 0.001. [mTOR—mammalian target of rapamycin; pmTOR—phospho-mammalian target of rapamycin; Akt—AKT serine-threonine kinase; pAkt—phospho-AKT serine-threonine kinase].

Western blotting was performed to determine whether prunetrin blocks the Akt/mTOR signaling pathway in Hep3B cells, and expression levels of all three proteins were observed. After treatment with PUR for 24 h, the phosphorylation of Akt and mTOR was significantly decreased in the higher dose (Figure 7) compared to the control cells. This result suggests that PUR can inhibit the cancer signaling pathway in Hep3B cells.

### 3.8. Effect of Prunetrin on MAPK Pathway

**Figure 8:**
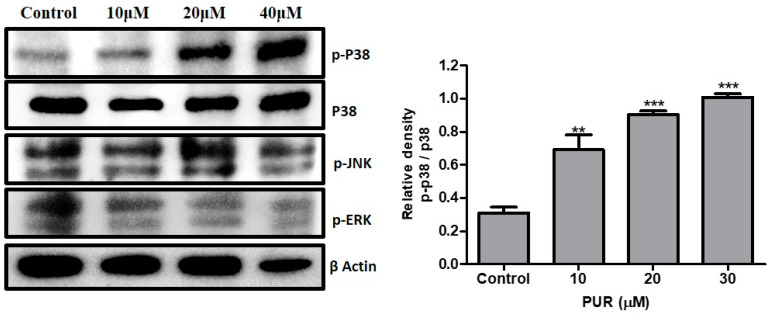
Effect of PUR on the MAPK signaling pathway. The cells were treated with PUR (10, 20, and 40 μM) for 24 h, and then isolated proteins were used for Western blotting. Densitometry was used to determine the expression levels using the SEM of three independent values. The results are presented as the mean ± standard error of the mean (SEM). ** = *p* < 0.01, and *** = *p* < 0.001. [ERK—extracellular-signal-regulated kinases; pERK—phospho-extracellular-signal-regulated kinases; JNK—Jun amino-terminal kinases; pJNK—phospho-Jun amino-terminal kinases].

The MAPK signaling pathway plays a significant role in cancer cell signaling. We further explored whether prunetrin affects MAPK proteins. The expression of MAPK signaling proteins in Hep3B cells was assessed after treatment with prunetrin for 24 h. As shown in Figure 8, phospho-p38 expression declined with the dose of the PUR concentration. In contrast, p-JNK and p-ERK remain unaltered after the treatment. This confirms that prunetrin induces cell cycle arrest by activating the p38 Hep3B cells. 

## 4. Discussion

Flavonoids have gained significant attention as potential anti-cancer agents due to their ability to regulate various signaling pathways involved in cancer development and progression. Previous studies have shown that flavonoids exhibit anti-cancer effects by inhibiting key enzymes involved in carcinogenesis and are found to modulate the expression of genes associated with cell cycle regulation, apoptosis, and angiogenesis in cancer cells. Moreover, dietary flavonoids have been demonstrated to be metabolized into products that inhibit cancer proliferation. Recently, studies reported that consuming foods with a high amount of flavonoids may lower cancer risk [23,24,25]. Additionally, research data indicate that flavonoid compounds tend to have the ability to inhibit cancer. Generally, an ideal compound should have the ability to inhibit cell proliferation by arresting the cell cycle, activating the cancer signaling pathway, and inducing the caspase cascade to promote apoptosis. It may have the ability to decrease mitochondrial potential [26].

Multiple studies have demonstrated that promoting or enhancing apoptosis in cancer cells can lead to their death and improve patient outcomes. Several potential targets for inducing apoptosis in cancer cells have been identified, including caspase activation [27], DNA damage response pathways, and signaling through death receptors [24]. One of the most important characteristics of cancer cells is their ability to evade apoptosis, a form of programmed cell death essential for maintaining tissue homeostasis. This ability is often due to mutations or dysregulation in the genes involved in regulating apoptosis pathways. As a result, targeting the apoptosis pathway has become an important focus in developing potential anti-cancer therapeutic agents. The manipulation of apoptosis pathways in cancer cells is a rapidly growing field that has demonstrated great potential for developing new anti-cancer therapeutic agents. In recent years, manipulating apoptosis pathways in cancer cells has emerged as a rapidly growing field with promising outcomes.

Our cell cytotoxicity (Figure 1A) and colony formation assay (Figure 1B) results showed that PUR has the ability to induce cytotoxicity and reduce the viability of cancer cells. The cell cycle is a highly regulated process that ensures the accurate replication and division of cells [28]. However, this process becomes dysregulated in cancer cells, resulting in uncontrolled cell proliferation and tumor growth. Dysregulated cell cycle progression is considered a hallmark of cancer, with evidence pointing to the important roles of dysregulated cyclins and cyclin-dependent kinases (CDKs) in many types of cancer [29,30]. CDKs belong to the serine/threonine protein kinase family and regulate cell cycle progression by phosphorylating specific substrates [31]. Our flow cytometry results indicate that PUR arrests the cell cycle at the G2/M phase (Figure 3A) and also dramatically decreases the expression of CDK1/CDK2, CDC25c, and Cyclin B1 proteins in a dose-dependent manner (Figure 3B), similar to previous data where the compounds polyphyllin D, apigetrin, and luteolin decreased the cell cycle protein expression dose-dependently [25,32,33]. 

Pieces of evidence show that the arrest of the cell cycle is habitually connected with the apoptotic cell death pathway [34]. Hence, flow cytometry analysis with Annexin V/PI was carried out. Our flow cytometry plot indicates apoptosis, with a high percentage of cells accumulating in the late apoptotic area (Figure 4A). Furthermore, we checked the cleavage of PARP and caspase 3. PARP is the key protein that initiates DNA damage and cascade activation. The cleavage of PARP is the main indication of apoptosis [35]. We observed the vast expression of cleaved PARP (Figure 4B) after treatment, which undoubtedly indicates caspase activation since cleaved PARP acts as a substrate for activated caspase 3. Our results revealed that PUR can induce apoptosis in hepatocellular cancer. Therefore, cell death, changes in cellular morphologies, cell cycle arrest, flow cytometry analysis, cleavage of PARP, and caspase 3 activation in Hep3B cells by PUR are confirmed to be apoptosis. 

We further explored the potential alterations that prunetrin may have on the molecular pathway of apoptosis. In general, apoptosis has two pathways: one is the death receptor pathway, and the other is the mitochondrial death pathway [36]. In the mitochondrial death pathway, Bcl2 family proteins play the main role in regulating apoptosis [37]. Bax’s role is to activate the caspase cascade with mitochondrial membrane permeability, but Bcl-xL can stop this progression, so Bcl-xL is an anti-apoptotic protein in nature [38]. Cytochrome C is a protein involved in ATP synthesis. However, during mitochondrial membrane destabilization, it releases to the cytoplasm of cells, triggers the caspase 9 activation, and is eventually involved in the apoptosis pathway [39,40]. Proofs from the literature have found that many compounds have decreased Bcl-xL expression and provoked the expression of bak, cytochrome C, and cleaved caspase 9 in breast cancer [41], liver cancer [42], and cervical cancer [43]. Similarly, we found that the cells treated with PUR decreased the Bcl-xL protein and increased the pro-apoptotic protein, Bak. Above the cytochrome C release, increased expression was also confirmed, and caspase 9 cleavage was also found (Figure 5).

The classic MAPK pathway is composed of extracellular signal-regulated kinases (ERKs), c-Jun NH2-terminal kinase (JNK), and p38 MAPK [44]. Studies have also shown that MAPK signaling cascades—including JNK, p38 MAPK, and ERK5—play essential roles, and abnormal activation of the MAPK pathway has been linked to the development and progression of cancer [45]. Numerous studies have confirmed that the MAPK pathway is overexpressed in various cancer cells and significantly contributes to their malignant potential. Therefore, abnormalities in MAPK signaling have become a significant target for developing novel cancer therapies that aim to block or regulate this pathway’s activation [13,46]. In particular, p38 MAPK is involved in the cell cycle arrest mechanism by inhibiting the cyclin and CDK proteins [47]. Interestingly, among the three MAPK proteins, phosphorylation of p38 was elevated significantly in a dose-dependent manner, while other MAPKs remained insignificant upon treatment. This result indicates that p38 MAPK is involved in the cell cycle arrest mechanism and the apoptotic pathway by PUR in Hep3B cells.

**Figure 9:**
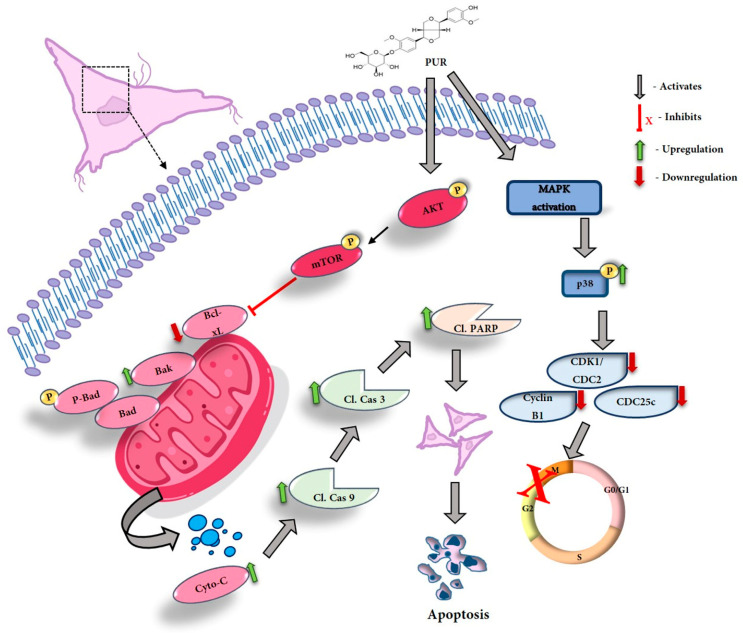
Schematic representation of PUR’s action on Hep3B cells.

Activation of the Akt/mTOR pathway in cancer cells has been implicated in various aspects of tumorigenesis. These include cell proliferation, survival, metabolism, angiogenesis, migration, invasion, and resistance to anti-cancer drugs [48], and Akt/mTOR activation is common in cancer cells. This leads to dysregulation of these pathways and enhanced cell proliferation [49]. The Akt pathway is also directly associated with caspase 9 and Bad activation [50]. Several studies have indicated that targeting Akt/mTOR pathways and their inhibition will result in an anti-cancerous effect [51]. Previous compounds like luteolin, pueraria flavonoids, and latifolin showed their ability to inhibit the Akt/mTOR pathway upon treatment [52,53,54]. Our data are similar to those of these compounds and show the potential to significantly inhibit the expression of both phosphorylated Akt and phosphorylated mTOR in a dose-dependent manner upon treatment with PUR. This is the first report on PUR’s effectiveness against Hep3B liver cancer cells. The overall mechanism of action is illustrated in Figure 9.

## 5. Conclusions

In conclusion, PUR was shown to have the ability to induce intrinsic apoptosis-mediated cell death in Hep3B cells. Furthermore, it was confirmed that PUR can arrest the cell cycle at the G2/M phase and activate the MAPK-p38 pathway. Also, this study reveals that the compound prunetrin can potentially inhibit cancer growth by activating the caspase cascade and inhibiting the Akt/mTOR pathways. In the future, strategies to improve the bioavailability and metabolic instability of flavonoids can be useful approaches to enhance flavonoid-induced therapeutic responses in cancer treatment. Furthermore, PUR can develop as an anti-cancer agent with further pre-clinical approaches and trials, including in vivo.

## Data Availability

The data used to support the findings of this study are available upon request from the corresponding author.
